# Health diplomacy: a new approach to the Muslim world?

**DOI:** 10.1186/1744-8603-10-50

**Published:** 2014-06-13

**Authors:** Mehrunisha Suleman, Raghib Ali, David J Kerr

**Affiliations:** 1 Ethox Centre, Nuffield Department of Population Health, Oxford University, Oxford, UK; 2 Faculty of Medicine, UAE University, UAE, Al-Ain, United Arab Emirates; 3 Nuffield Department of Clinical and Laboratory Sciences, University of Oxford, Oxford, UK

## Abstract

Three years ago, the Lancet’s frontispiece stated “Health is now the most important foreign policy issue of our time” and last year, the Director-General of WHO, Margaret Chan, in her opening address, to the Executive Board at its 132^nd^ Session said “health diplomacy works”. The nascent field of health diplomacy provides a political framework which aims to deliver the dual goals of improved health in target populations and enhanced governmental relations between collaborating countries. Any government that offered tangible health improvement as a component of aid to a nation with whom they wished to develop stronger diplomatic links would have an advantage in developing a deeper relationship with its citizens.

Here we suggest several different mechanisms through which such links could be developed or enhanced, including: provision of relevant health solutions, applied research, cultural alignment and the development of collaborative networks. The Islamic tradition promotes the practice of medicine as a service to humanity. Physical and spiritual wellbeing are intimately related in popular Muslim consciousness. Thoughtful Health Diplomacy therefore has the potential to bridge the perceived divides between Western and predominantly Muslim nations.

## Background

Christian physicians and surgeons of the 19^th^ and early 20^th^ centuries laid many of the foundations for modern medicine in Africa, China, India and the Americas. The most famous of these, David Livingstone and Albert Schweitzer had almost mythic status, as missionary martyrs, explorers, anti-slavery crusaders and physicians. Livingstone and Schweitzer set the historical precedents for Health Diplomacy and paved the way for subsequent developments in colonial and post-colonial policy in which health played an important role. Schweitzer, of course, was awarded the Nobel Peace Prize, as were the Red Cross and Médecins Sans Frontières, further emphasising the link between the practice of medicine and the quest for peace. Since then, we have seen the rise of global philanthropic institutions, notably the Bill and Melinda Gates Foundation, which wield enormous strategic and financial clout [1].

The 20^th^ century also witnessed the greatest betrayal of the Hippocratic ideal, of the physician as a guardian of health, by doctors such as Josef Mengele who supervised the entire process of the medical mass murder of millions of Jews, communists and other “racial undesirables”, as well as the earlier sterilisation and euthanasia of its own citizens. More recently, U.S doctors have been complicit in torture in Iraq, Afghanistan and Guantanamo Bay [2] and the prominent role of Ayman al-Zawahiri, an Egyptian surgeon, in the terrorist activities of Al-Qaeda, and of a British Iraqi doctor in the terrorist attack on Glasgow airport and Central London in 2005 have reminded us that such betrayals are not confined to history.

The latter is, a travesty of Islamic Medicine, whose Hippocratic-Oath- equivalent (based on the teachings of the Quran and sayings of Prophet Muhammad) exhorts doctors to serve “all of mankind, poor or rich, literate or illiterate, Muslim or non-Muslim, black or white with patience and tolerance, with virtue and reverence, with knowledge and vigilance.” [3] There are many verses in the Qur’an and sayings of Prophet Muhammad (peace be upon him) relating to medicine, mentioning the virtues of treating the sick and the great rewards for relieving suffering and ill-health. For example, the Quran says, “whoso saveth the life of one, it shall be as if he had saved the life of all mankind” [4] and the Prophet stated that “there is a reward for serving any animate (living being)” [5].

At the time of the Prophet’s death, in 632 AD, he had successfully united the numerous tribes of Arabia into a powerful nation. The latter expanded from the Atlantic Ocean on the West, to the borders of China on the East [6]. The Arabs did not destroy but rather assimilated the cultures and knowledge of the people they ruled. The birth and growth of the Islamic state led to a flourishing of scholarship, particularly in the field of medicine. Al Razi (in Latin: Rhazes) who died in 923–24 AD was said to have written 100 books on medicine alone. His famous monograph on smallpox and measles “Kitab fi al-jadari wal hasba” (De variolis et morbilis; de peste, de pestilentia) [7] impacted the research and practice of physicians hundreds of years later.

Ibn Sina (in Latin: Avicenna) wrote the enormous medical encyclopaedia the Qanun (Canon), which remained a key reference not only within the Islamic state but the whole world. He was honoured with the title the “prince of physicians” and his book was the most widely studied work of medicine from the 12^th^ to the 17^th^ century. These and many other Muslim scholars, such as Al-Zahrawi (Abulcasis) and Ibn Juljul of Cordoba, from the medieval period, are renowned for their influence on medicine, which has had a global and lasting impact. Such scholars studied philosophy and medicine concurrently thus establishing the epistemological link between medicine and religion [8,9]. Additionally, the linguistic relationship employed by such scholars, between philosophical and medical terms such as soul, knowledge and God in theology, and spirit, soul and body in Galenic medicine, interwove the two disciplines. It led to the intrinsic connection between religion and medicine in Islam [10]. Thus in the medieval period, within all three Abrahamic faiths, not only Islam; philosophy and medicine were practiced simultaneously. It is for this reason that throughout the Islamic Empire the term hakim was used to refer to both physicians and philosophers [8]. Although the epistemological relationship between religion and medicine relates to traditional medicine, the central feature of relieving suffering is common to both contemporary and traditional means of healing.

At the time, Islamic medicine represented an international and intercultural scholarly exchange, across borders of empires, fostered by the Islamic rulers in Arabia, Baghdad, Damascus, India and Spain. Such cross border sharing of knowledge, expertise, resources and the shared vision of human flourishing is what we see as a potential outcome of contemporary “health diplomacy”.

Thus the teachings of Islam and its observance by Muslims during the formative period of the religion promote the practice of medicine as a service to humanity. Physical and spiritual wellbeing are intimately related in popular Muslim consciousness. We propose, therefore, that thoughtful Health Diplomacy has the potential to bridge the perceived divides between Western and predominantly Muslim nations. Here we suggest several different mechanisms through which such links could be developed or enhanced, including: provision of relevant health solutions, applied research, cultural alignment and the development of collaborative networks.

### Mechanisms for developing and enhancing “Health Diplomacy”

Three years ago, the Lancet’s frontispiece stated “Health is now the most important foreign policy issue of our time” and last year, the Director-General of WHO, Margaret Chan, in her opening address, to the Executive Board at its 132nd Session said “health diplomacy works” [11]. The nascent field of health diplomacy provides a political framework, which aims to deliver the dual goals of improved health in target populations and enhanced governmental relations between collaborating countries. Any government that offered tangible health improvement as a component of aid to a nation with whom they wished to develop stronger diplomatic links would have an advantage in developing a deeper relationship with its citizens.

There are several different mechanisms through which this could be accomplished e.g. provision of generic funding, ensuring supplies of essential drugs, capital investment in hospitals or equipment and training health professionals. There have been remarkable investments in Sub-Saharan Africa, South America and South-East Asia, often focussed on eradication of specific diseases e.g. AIDS, malaria and TB, but thus far, relatively little support for general health infrastructure, training and education, or the chronic disease burden of developing countries [12].

The imperatives of globalisation, brought sharply into focus by the recent swine flu outbreak, have focussed most efforts of health diplomacy on cooperative, international efforts to deal with trans-border health threats e.g. SARS, Swine Flu or of combating the threat of bioterrorism, defending national borders against incursion by pathogens. e.g. anthrax. There is nothing new in this - in the mid-nineteenth century, Virchow, espoused that social, economic and political factors were more important than biological causes in a typhus epidemic in 19^th^ century Prussia, commenting that “Medicine is a social science, and politics nothing but medicine on a grand scale” [13].

With increasing consideration being given to the use of “soft power” to achieve diplomatic aims, we believe that there is an opportunity to extend the scope of health diplomacy and enhance foreign policy practice by encompassing the following additional features:

1. **Relevant Health Solutions:** Strengthen those aspects of health infrastructure which the recipient nation deems important. With one child dying every five seconds from hunger and malnutrition [14] providing food for the hungry and health education to the lay public could arguably be deemed more important and relevant in some countries than medicinal drugs. It is vital to avoid the imposition of health “solutions” which may be irrelevant, e.g. construction of a hospital which is never staffed; or equipment which is never “switched on”, or unaffordable. The past twenty years have seen a four-fold increase in funding for development assistance for global health [1]. That period has also seen a change in the balance of power and priority setting between the various donor organisations with an explosion in the number of players on the health pitch. An interesting editorial in the Lancet had suggested a note of caution over the potential of those vertical Global Health Initiatives to distort the true health priorities of the recipient nations and distract them from more sustainable long-term goals e.g. strengthening of primary care [15]. Collectivism rather than competition is needed if the various donor organizations’ efforts are to be effective. The experience of “Hajj Medicine” in 2009 provides an unprecedented example of not only a relevant but also an essential health solution, achieved through collaborative efforts between the US and the Kingdom of Saudi Arabia (KSA). The Hajj is a pillar of Islam which requires believers of mature age and sufficient means to journey to Mecca. It is an endeavour of every Muslim to undertake this journey and the Hajj pilgrimage in recent years has attracted over 2.5 million people, and this figure is set to rise with the rising global population. As the largest mass gathering in the world, the Hajj presents unique Public Health challenges affecting residents of almost every continent across the globe. The H1N1 outbreak in 2009 posed an immense challenge to the local authorities requiring a collaborative effort between the US Centre for Disease Control (CDC) and Ministry of Health of the KSA. Their teams successfully planned and employed robust surveillance strategies [16]. Their work surpassed all international expectations as it prevented a much anticipated and feared post-Hajj spike in H1N1 cases. Relevant health solutions for the nascent field of “Hajj Medicine” may provide a powerful means of harmonizing relations between Western and predominantly Muslim nations.

2. **Applied Research:** Translation of modern biomedical research into deliberately cost-effective health interventions for poorer nations, perhaps by using open access science and avoiding the pitfalls associated with the protection of intellectual property is a key step to achieving equitable global health as well as diplomatic aims [17]. There is increasing awareness that major disease burdens can contribute to the weakening of state capacity and the destabilisation of states is closely associated with the threat of weak or failed states [18]. Controversies surrounding the limitations to equitable access to the influenza vaccine during the avian influenza A (H5N1) and the pandemic influenza A (H1N1) have led to defiance of international protocols. In 2008, Indonesia’s then-health minister refused to share its H5N1 samples or report incidences of the disease, insisting that she wanted guarantees from the developed world and drug manufacturers that developing countries would be able to access affordable vaccines after donating their samples [19]. Methods to overcome such diplomatic and global health challenges can include greater transparency, agreed benefit sharing protocols and, as suggested above, local empowerment through the strengthening of local capacity for research and development [20].

3. **Cultural Alignment:** Applying health diplomacy in a manner which is ethically, culturally and politically attuned is imperative for successful adoption and implementation of strategies and interventions. This does not mean that we back off from areas of controversy, but rather work to expand existing political and cultural frameworks to accommodate sustainable change in the longer term. There are excellent examples of this around the use of male/female condoms for HIV/AIDS protection in Sub-Saharan Africa and other efforts to promote gender equality and empowerment of women in the field of training healthcare professionals. Muslim countries, previously considered protected from HIV/AIDS due to religious and cultural norms, are facing a rapidly rising threat [21]. The common response from policy makers in Muslim countries, for HIV prevention, has been a focus on abstention from illicit drugs and sexual practices. Condom use and the provision of clean needles had been discouraged as it was considered that this would lead to sexual promiscuity and illicit drug use. However, more recently, in countries where HIV/AIDS is rapidly rising, such as Uganda [22] and Indonesia [23] Muslim scholars have taken a more flexible stance and justified the use of condoms and clean needles as a short-term means of ensuring the sanctity of life. The principle of saving life is mentioned in the Quranic verse above and under a state of emergency, sanctity of life, overrides the discouragement of condom use [23]. Such cultural adaptation has required extensive work by local and international health professionals with the religious leaders as key collaborators to ensure public acceptance and adoption of the prevention measures.

4. **Collaborative Networks:** Building strong educational and research links between networks of collaborating physicians and healthcare professionals, who see the pursuit of peace as a rational public health consequence of their involvement in the practice of medicine may be an ideal vehicle for delivering Health Diplomacy. In his Cairo speech to the Muslim World, President Obama made a clear commitment, to expand exchange programmes and increase the degree of interaction between Western and predominantly Muslim nations [24]. Establishing networks of physicians, who would cooperate through exchange of knowledge, who would be prepared to mobilise and offer help in times of need, and bear accurate witness, if needs be, to the atrocities inflicted by others in times of conflict, would be a logical correlate of Obama’s initiatives.

Indonesia, the world’s largest Muslim majority country was, until recently, largely inward and suspicious of foreign, particular Western intervention. In 2010, an Indonesian health minister, citing allegations of espionage, closed down the U.S. supported Naval Medical Research Unit (NAMRU-2) which had been conducting biomedical research in Indonesia since 1970 [25]. The example of H5N1 cited above highlights another instance of Indonesia’s lack of trust in existing collaborative networks.

However, over the past 4–5 years with a burgeoning economy the country has become more active in regional and international diplomacy [26]. Indonesia’s health minister has been appointed as chair for the Global Fund to fight AIDS, TB and malaria, the country’s president is co-chairing a high-level panel advising the UN secretary general on the global development agenda, beyond 2015. The country will also be hosting a conference of Asia-Pacific finance and health ministers to discuss potential funding mechanisms for the provision of universal health coverage in the region [26]. Collaborative efforts involving the rotation or sharing of leadership for global initiatives is a means of improving and increasing transparency, facilitating local awareness of global efforts and dispelling myths and suspicions. The latter mechanisms may thus allow otherwise hesitant nations to take on the responsibility of health diplomacy and the successful shared ownership of global health efforts.

Successful collaborative efforts between Western and predominantly Muslim nations include the response to an outbreak of a viral illness among pig farmers in Malaysia. Between 1998 and 1999 there were 101 recorded deaths in Malaysia and 1 in Singapore. Close collaboration between Malaysian and Australian health authorities as well as the WHO Collaborating Centre led to the successful control of the spread of the disease. Australia organised a range of experts from human and animal health agencies to support Malaysian diagnostic and control efforts. This occurred at a time when the two countries were facing diplomatic tensions during Prime Minister Mataher’s incumbency [27].

Another related priority within the establishment of collaborative networks is the recognition of the need to rebuild health systems in Iraq, Afghanistan and other conflict and post-conflict areas. This will require both intellectual and material investment by neighbouring and international states to help reconstruct a sustainable means of providing healthcare, with the latter also forming part of the wider counterinsurgency efforts [28].

### Organisation of the Islamic Conference: platform for collaboration?

Within the Muslim world, the emergence of a number of middle-income countries with influence and interest in global health opens a multitude of opportunities for lasting collaboration. Evolving economies such as Malaysia, the UAE and Qatar occupy a complex regional and international role. Malaysia helped establish the Organisation of the Islamic Conference in 1969. It is comprised of 57 countries with headquarters in Jeddah, Saudi Arabia. The organisation was formed to promote cooperation between countries with an Islamic identity. The organisation’s mandate has been extended to embrace cooperation in the matter of health also [29].

The OIC may consider developing its “health programme” to function as a conduit through which cooperation and collaboration can occur between Western and predominantly Muslim nations. More specifically it may consider allocating resources to ascertain emerging national and regional health priorities and be the channel through which collective representation and action can occur. A clear means of communication, planning and delivery of health may provide a means of improving health and diplomatic outcomes for both governmental, non-governmental global foundations and also state sponsored intergovernmental relations. Working beyond state, regional and institutional boundaries is the only means through which successful global health and diplomatic outcomes can be achieved.

## Conclusions

If the concept of health diplomacy is developed thoughtfully and rigorously by Foreign Service and medical leaders, it may help to avert conflict, augment peace, support altruism and economic progress and cement sustainable international cooperation. Perhaps one immediate area in which to test this hypothesis might be to strengthen ties between Western and predominantly Muslim nations, especially in this resurgent Arab Spring. US efforts in Aceh, following the tsunami, yielded immense support from the local population. A poll by the Heritage Foundation found “the first substantial shift of public opinion in the Muslim world since the beginning of the US global war on terrorism” when support for the US rose from 36 per cent to 60 per cent in the world’s largest Muslim country [30].

## Competing interest

The authors declare that there are no competing interests.

## Authors’ contributions

MS helped draft, update and edit the manuscript. RA helped draft and edit the manuscript. DK developed the concept for the article and helped draft and edit the manuscript. All authors read and approved the final manuscript.
